# Memories that last: epigenetic regulation of cold stress response prepares plants for subsequent stress events

**DOI:** 10.1093/plphys/kiae579

**Published:** 2024-10-29

**Authors:** Aida Maric

**Affiliations:** Assistant Features Editor, Plant Physiology, American Society of Plant Biologists; CIBSS - Centre for Integrative Biological Signalling Studies, University of Freiburg, Freiburg 79104, Germany; Plant Environmental Signalling and Development, Institute of Biology III, University of Freiburg, Freiburg 79104, Germany

Plants exposed to sublethal doses of stress can create a “molecular memory” signature, allowing them to be more resilient to future stresses (Bruce et al. 2007; Lamke and Baurle 2017). Molecular memory signatures are created through changes at different levels, from metabolomic to transcriptional. The transcriptional memory changes that plants undergo in the face of different biotic and abiotic stresses has become an expanding research field (Charng et al. 2023).

Transcriptional memory genes have different expression patterns in the primed plants—which have experienced and survived at least 1 sublethal stress—compared with the naïve plants, which had never encountered the stress. Previous studies described 2 types of transcriptional memory: type I and type II memory (Fig. 1A). Type I memory is generally characterized by maintenance of altered gene expression after the first stress ends. Type II memory is marked by gene expression that goes back to basal levels after the first stress but has an enhanced or repressed expression upon the second stress compared with plants experiencing the stress for the first time.

**Figure 1.**
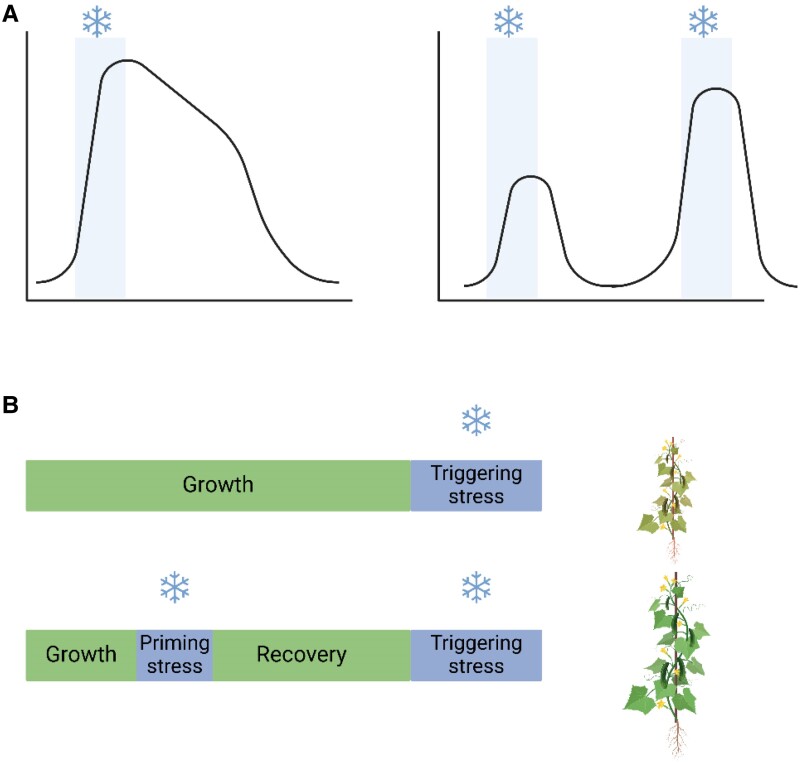
**A)** Expression patterns of type I (left) memory genes. The expression stays altered even after the stress is over. Type II (right) memory genes where expression levels go back to basal levels after the first stress is over but have enhanced expression levels upon the second stress. **B)** Naïve plants exposed only to the triggering stress have lower tolerance to cold compared with primed plants that experienced at least one previous cold stress. Created using BioRender.

Plant stress memory research often focuses on the changes occurring during the second stress, but in a recent article published in *Plant Physiology*, Di et al. (2024) explored the transcriptional changes that occurred during the recovery phase—after the first stress. The authors showed that cucumber seedlings that underwent priming cold stress have better tolerance upon the second cold stress compared with naïve plants (Fig. 1B). To explore the basis of this phenotype, they explored in detail the transcriptional changes during the recovery phase in the type I and type II memory genes. Using gene ontology (GO) analysis, the authors showed that type I memory genes responsible for protein synthesis were upregulated, while GO terms related to protein processing were downregulated. This is in line with previous studies that reported how plants under stress invest in protein synthesis (Liu et al. 2014). Therefore, the authors suggest that transcriptional memory of cold stress is associated with increased protein synthesis and decreased protein processing and folding.

Somatic memory can be controlled by epigenetic state changes in the form of histone mark modifications, and such poised epigenetic states can alter gene expression. Di et al. (2024) reported a detailed investigation of the genome-wide epigenetic changes regulating differences between different types of memory genes after cold stress in cucumber plants. Specifically, they focused on the histone modification that leads to gene activation: histone 3 lysine 4 trimethylation (H3K4me3). Using chromatin immunoprecipitation-Seq of primed and naïve plants, they showed that H3K4me3 stays accumulated at part of the memory genes of primed plants during the recovery period. They hypothesized that accumulation of the active H3K4me3 mark leads to a faster transcriptional response upon second stress in the primed plants and therefore constitutes an epigenetic memory.

In line with these findings, Di et al. (2024) identified a member of the cucumber respiratory burst oxidase homologue (RBOHs) family—CsRBOH5.1—to be a regulator of the transcription during the recovery phase. Using CRISPR-generated mutants, the authors showed that CsRBOH5.1 regulates the H3K4me3 deposition at different memory genes during the recovery phase. Consequently, the memory signature of different genes was abolished in *Csrboh5.1* mutant. These experiments provide a new candidate for regulation of memory formation after cold stress. Furthermore, the RBOHs catalyze the formation of H_2_O_2_ (Otulak-Kozieł et al. 2019). The levels of H_2_O_2_ were previously used as a readout for stress memory formation upon heat or salt stress (Sun et al. 2018; Di et al. 2022). Di et al. (2024) show that induced expression of *CsRBOH5.1* during recovery phase after the cold stress leads to increased levels of H_2_O_2_ and the formation of cold stress memory.

Dynamic regulation of H3K4 trimethylation is one of the most studied mechanisms known to regulate stress memory. While genome-wide studies are useful to identify possible candidates regulating memory formation, they lack the precision and direct proof for causal evidence. Future studies may aim for regulating H3K4me3 at specific loci using epigenome editing, which may lead to biotechnological breakthrough allowing tight regulation of memory formation, even without the priming stress and uncouple cause and effect between specific histone marks and transcriptional activity. Currently, different abiotic stresses from cold to drought or flooding cause big agricultural losses and food insecurity worldwide. Regulation of transcriptional changes by depositing and/or erasing specific histone modifications can lead to rescue of large portions of crops. One can envision a future where we have an epigenetic toolbox allowing us to have increased control of intensity, speed, and duration of transcriptional changes and stress response.

## Data Availability

No data associated with this article.
